# The perspective and experiences of significant others on electroconvulsive therapy

**DOI:** 10.3389/fpsyt.2025.1575088

**Published:** 2025-04-09

**Authors:** Pieter-Jan Geerts, Souad Abihi, Nele Van De Velde, Chris Baeken, Gilbert Lemmens, Sofie Verhaeghe

**Affiliations:** ^1^ Department of Psychiatry, AZ Groeninge, Kortrijk, Belgium; ^2^ Department of Head and Skin - Psychiatry and Medical Psychology, Ghent University, Ghent, Belgium; ^3^ Department of Psychiatry, Ghent University Hospital, Ghent, Belgium; ^4^ Ghent Experimental Psychiatry (GHEP) Lab, Faculty of Medicine and Health Sciences, Department of Head and Skin, Ghent University, Ghent, Belgium; ^5^ Neuroprotection and Neuromodulation Research Group (NEUR), Center for Neurosciences (C4N), Vrije Universiteit Brussel (VUB), Brussels, Belgium; ^6^ Center for Care and Cure Technology (C3Te), Department of Electrical Engineering, Eindhoven University of Technology, Eindhoven, Netherlands; ^7^ Department of Psychiatry, Universitair Ziekenhuis Brussel (UZ Brussel), Brussels, Belgium; ^8^ University Centre for Nursing and Midwifery, Department of Public Health and Primary Care, Ghent University, Ghent, Belgium; ^9^ Department of Nursing, VIVES University College KULeuven, Roeselare, Belgium; ^10^ Faculty of Medicine and Life Science, Hasselt University, Hasselt, Belgium

**Keywords:** electroconvulsive therapy, significant others, decision-making, hope, stigma, perspective, experiences

## Abstract

**Introduction:**

Electroconvulsive therapy (ECT) is an essential but often controversial treatment in psychiatry. While existing research focuses on patient outcomes, the perspectives of significant others (SOs) remain underexplored. They play, nevertheless, a crucial role in decision-making, treatment adherence, and post-treatment evaluation. To better understand their perceptions, challenges, and support needs, this study aims to explore the lived experiences of SOs and ECT.

**Methods:**

A qualitative phenomenological approach was employed using semi-structured interviews with nine SOs of patients who underwent ECT. Thematic analysis was conducted using Braun and Clarke’s framework, and data were analyzed using the NVivo software.

**Results:**

Before ECT, SOs experienced a significant emotional burden, describing their lives as unlivable due to the patients’ severe illness. The decision to start ECT was marked by feelings of responsibility and fear but also driven by hope. During ECT, SOs closely monitored treatment effects and side effects, balancing improvements against challenges such as memory loss and fatigue. The psychiatrist played a central role in shaping perceptions and instilling hope. During the maintenance phase, SOs faced logistical challenges and stigma but aimed to integrate ECT into daily life while supporting patient autonomy.

**Conclusion:**

This study highlights the complex role of SOs in ECT. Unlike previous studies that have focused on caregiver burden, it emphasizes the role of hope in decision-making and treatment adherence. SOs value transparent communication from psychiatrists and seek structured support systems to navigate practical and emotional challenges. Stigma remains a significant barrier to open discussion and social integration.

## Introduction

Electroconvulsive therapy (ECT) is an essential treatment in psychiatry (1). Despite its importance, it remains a controversial and sometimes misunderstood treatment (2, 3). Fears surrounding the use of electrical impulses on the brain, negative representations in movies and games, and the primitive practices of the past have contributed to uncertainty that extends beyond that of the majority of other therapies (4–6). When ECT is proposed, patients and their significant others (SOs) often voice their fears. The term “significant other” is used in this study to include individuals who have a meaningful personal relationship with the patient, regardless of legal or caregiving obligations. In existing research, terms such as “carer” and “caregiver” are commonly used. While some SOs appreciate this label as they recognize their supportive role in the patient’s care and adherence to treatment, others reject them, as they may imply a formalized caregiving role that does not align with their personal relationship, as it may create division between the caregiver and the person receiving care (7). Because patients eligible for ECT are often severely ill and may have limited capacity to give informed consent, SOs play a crucial role in the decision-making process. A recent review found that SOs generally have positive attitudes toward ECT and report high levels of satisfaction with treatment (8). However, many SOs express a need for more comprehensive information regarding side effects, prognosis, and long-term impact. The majority of studies of SO perspectives have relied on quantitative measures such as surveys, limiting the depth of understanding of their experiences (8). A qualitative exploration could enhance the role of ECT practitioners in guiding both patients and their SOs through the decision-making process, which not only is crucial at the start of the treatment but also requires ongoing assessment throughout the ECT treatment. As maintenance ECT (M-ECT) is increasingly recognized as a vital strategy to mitigate the high risk of relapse (9–12), the decision to continue or discontinue treatment becomes an ongoing concern for both patients and SOs. Despite this, little is known about how SOs perceive their role in these decisions and how they evaluate the benefits and risks of ECT over time. This study aimed to explore the lived experiences of SOs during the period of ECT. The secondary objectives were to identify the support needs of SOs and to assess the role of psychiatrists in shaping their perspectives throughout the treatment process.

## Methods

### Study design

To gain insight into the lived experiences and perceptions of SOs on ECT, a qualitative phenomenological approach was used (13). Semi-structured interviews were conducted with SOs, and the data were analyzed thematically using the framework developed by Braun and Clarke (14). The COnsolidated criteria for REporting Qualitative research (COREQ) checklist was utilized to ensure comprehensive and transparent reporting of the findings.

### Setting

Patients were recruited from the ECT unit of a large general hospital. This unit treats in- and outpatients with ECT, along with referrals from other general and psychiatric hospitals in the area. Both acute ECT and maintenance ECT were provided.

### Sampling and participants

To capture a broad range of experiences and perceptions, purposive sampling was employed. Participants were selected to ensure diversity in demographic characteristics (e.g., gender and age) and treatment-related factors (e.g., number of ECT sessions and phase of ECT). Although this study did not aim to compare perceptions between acute ECT and M-ECT, it included SOs from both groups. Patients were provided with an information letter about the study and were invited to personally nominate SOs to participate. SOs who expressed interest were contacted by telephone, and the study was explained in detail. Informed consent was obtained before conducting interviews. Notably, all approached patients and their SOs agreed to participate.

Participants were eligible if they were Dutch-speaking adults identified by patients as their primary SOs and had had experience with ECT within the past year.

### Data collection

From September 2022 to June 2023, SOs participated in a single interview one-on-one with an SA, an advanced practice nurse in mental health. Interviews were conducted based on the preference of the participants, at their home (n = 5) or in a meeting room in the hospital (n = 4), to make them feel at ease and to be relevant to their daily lives (15, 16). There was no relationship between the participants and the researcher prior to the interview, and participants knew the researcher’s occupation and affiliation.

Each interview began with an open-ended question to introduce the focus without directing it toward a specific theme. The opening question was, “I understand that you are a significant other for someone who is/was treated with ECT. Can you describe how you experience(d) that?” Follow-up questions had the purpose of gaining a deeper understanding of what the participants were bringing to the interview. They were formulated as, e.g., “Can you tell me something more about it, can you give an example, what did that mean for you?” All interviews were audio-recorded and transcribed verbatim. The average duration of the interviews was 120 minutes. Nine participants were included. The mean age was 68.5 years, and the mean number of ECT sessions was 78. The respondent characteristics are listed in **Table 1**.

**Table 1:** Characteristics of relatives.

	Total n = 9
Female respondents	n = 5
Age SO	
Age 18–35	1 (11.1%)
Age 36–55	0
Age 46–55	0
Age 56–65	2 (22.2%)
Age 66–75	3 (33.3%)
Age 76–87	3 (33.3%)
Diagnosis of patient	
Difficult to treat depression	3 (33.3%)
Psychotic depression	5 (55.6%)
Catatonia	1 (11.1%)
Relation to patient	
Parent	3 (33.3%)
Partner	3 (33.3%)
Friend	1 (11.1%)
Child	1 (11.1%)
Cohabiting with patient	6 (66.7%)
Timing of interview to last ECT in days	
<7	3 (33.3%)
7–14	4 (44.4%)
14–21	2 (22.2%)
ECT course phase	
Maintenance ECT	6 (77.8%)
Continuation ECT	1 (11.1%)
During acute course	1 (11.1%)
After acute course	1 (11.1%)
Number of ECT sessions	
0–10	1 (11.1%)
10–20	1 (11.1%)
20–50	4 (44.4%)
>50	3 (33.3%)

SO, significant other; ECT, electroconvulsive therapy; d, days.

### Analysis

The research team consisted of an advanced practice nurse (SA) experienced inpsychiatric care, a psychiatrist (PG) experienced in ECT and geriatric psychiatry, a professor and PhD nurse (SV) experienced in mental health and qualitative research methods, a psychiatrist experienced in ECT (NVDV), and an associate professor and psychiatrist (GL) experienced in systemic psychotherapy, liaison psychiatry, and qualitative research collaborated. The diversity in backgrounds broadened and deepened the analyses (17). SA conducted the interviews. The interviews were critically revised by SV to avoid biased, influencing, or closed questions and lack of exploratory and in-depth questions. The first analysis of the data was conducted by SA, PG, and SV. They read and reread the interviews independently. This enabled them to immerse themselves in the data and to identify similarities, differences, and patterns. Discussions and reflections formed the basis for the coding. In the next step, the SA coded the transcripts in the NVivo11 software. Critical reflections on the coding were discussed with PG and SV, code categories were created, and themes were conceptualized. The researchers contextualized the findings, weaved together the analytical narrative, and added data extracts to inform their findings. The findings were then discussed with the other authors (GL and NVDV). Based on their questions and critical appraisal, the analyses were broadened and deepened. Data saturation was reached for all reported themes.

### Ethics

The study was approved by the ethics committee of AZ Groeninge, Kortrijk. All participants provided signed informed consent prior to the study. Anonymity and confidentiality were maintained throughout the entire research process. All interviews were anonymized by removing identifiable details from the transcripts. Data were securely stored and encrypted, ensuring full confidentiality throughout the research process.

## Results

### Before ECT

#### “Life is no longer livable”

Throughout the interviews, SOs consistently reported a long trajectory before the first mention of ECT. They described a journey marked by increasingly more disabling symptoms of the patient and a significant impact on daily life. The majority of SOs mentioned psychiatric hospitalizations, psychotherapy, and psychopharmacologic strategies before ECT, which often resulted in temporary or no success. As the illness became central to the SOs’ lives, they began to reverberate with the illness. When symptoms improved, they felt the positive aspects and optimism. When the patient’s symptoms worsened, they experienced increased stress, negatively impacting their own lives. The SOs appeared to have no control over the illness and, consequently, over their own lives. Their lives appeared to be entirely dictated by the illness, and they expressed a sense of powerlessness over it. During this process, they adjusted their expectations about life and what they wanted from it. They pushed boundaries, sought solutions, and tried to adapt.

“I started working part-time so that I can continue to combine caregiving with a job. Although it is financially very difficult and I don’t know how we will cope, there is no other option right now”

Despite intensive treatment and therapy, the illness kept demanding more. It took a central place in the life of the patient and the SO. It gradually made their lives unrecognizably different than before.

“We become isolated, can’t meet with friends anymore and even can’t see the children in the weekends or go for a bike ride together as we did for so many years.”

By the time ECT was considered, SOs indicated that life no longer felt livable. It became a matter of survival, with daily struggles to support the patient to accomplish even the most basic tasks. Essential activities like getting up, dressing, and even eating required special effort from the SOs. Social contacts were absent, and SOs often did not find the time and possibility to fulfill their basic needs. Activities such as eating, taking a shower, and going to the supermarket have to be planned and must be done in a rush.

“She doesn’t accomplish anything anymore, whereas she used to be a vibrant woman, managing the household, having a responsible job. Now, having the children around is already too much.”

The SOs’ energy was depleted, and the limit of what is livable was reached. SOs described it as “life is not life anymore”. In this context, they indicated that the decision to start ECT is not really a choice. It appears to be the only possibility to get out of an unbearable situation that keeps worsening, and there appears to be no alternative.

#### The weight of responsibility and the perspective of hope in the decision to start ECT

In comparison to medication or psychotherapy, SOs saw ECT as a more radical treatment. The effect of electricity on the brain appeared to be more dangerous to them than the effect of chemical products.

“You have the image in your head of someone shaking and having a kind of epileptic insult. A pill is just a pill. You take it and … finished.”

ECT is a treatment that SOs did not have in mind when they first heard of it. Several SOs indicated that when ECT was introduced as a possible treatment, it induced much uncertainty and anxious feelings. SOs regarded ECT as an old and rather inhumane therapy, and some SOs were surprised that it was still being used. Since ECT was not really known to the SOs, they had many questions about the procedure.

When giving consent for ECT, SOs reported feeling fully responsible for the decision, as patients were sometimes unable to provide consent themselves due to their illness. SOs indicated that it was even difficult to have a conversation with the patient about (starting) ECT. SOs who were legally required to provide formal consent and whose loved ones were able to consent indicated that this feeling of responsibility put a burden on their shoulders and induced fear. Because of the responsibility they felt, support for this decision was often sought from another family member or friend.

“Imagine if something were to go wrong; I did make the decision for my mom.”

#### The psychiatrist and his/her role in decision-making and trust

SOs all indicated that the psychiatrist is pivotal in the process that they go through, especially in the process of decision-making. Information regarding ECT, more specifically on the effects and the side effects, is important, and the psychiatrist is the first and most important source to provide this information. While brochures, internet resources, and explanations from nurses and other staff provide helpful knowledge about the treatment and practical aspects, SOs perceived the psychiatrist as the person who has answers to their most important and pressing questions and concerns. A psychiatrist who demonstrates interest in understanding the patient’s specific situation, acknowledges the unique needs, and expresses genuine concern fosters a strong sense of trust in SOs. This trust reassures SOs that the patient is receiving individualized care and is not merely one of many being treated. SOs emphasized that confidence in the psychiatrist alleviated fear and uncertainty, making the decision to proceed with ECT more manageable. A question that SOs often (wanted to) ask was what the psychiatrist would decide if it was, e.g., his/her mother, partner, or brother. If the psychiatrist answered that he/she would decide to start the treatment, SOs felt strengthened in their decision to start ECT. When a psychiatrist stated that starting ECT was a good decision, it relieved the SOs of the burden of responsibility.

#### The psychiatrist and his/her role in instilling hope and shaping perspectives

By receiving information about ECT and its effects, hope is induced in SOs. When the psychiatrist shared his/her experience with ECT and its effects on other patients, this reassured them and introduced hope. Afterward, they mainly remembered positive remarks from the received information. Concerning the effect of ECT, words such as significant, substantial, successful, and improvement were remembered by the SOs. Side effects and the knowledge that ECT is not always successful were addressed and considered important. However, in weighing their decision, SOs gave less weight to the possible side effects, as hope for a life that is more livable was more prominent and necessary to make the decision for ECT.

“After I asked, the psychiatrist said that he would decide to start ECT for his partner. I was really sure then, that ECT was ok and the only option to get out of the misery.”

“The psychiatrist has seen good effects of ECT and saw patients as my wife who couldn’t accomplish anything anymore and who recovered to function normally again. Although I am rather careful, hearing this from someone who knows and who has seen the recovery in many patients, gives hope. It gives you the courage to try it and to go on enduring and hope that ECT will bring relief.”

“How will the future be? I don’t know. We hope for ECT to bring change. It can’t remain as it is now.”

### During ECT

#### Monitoring and balancing effects and side effects

During ECT, SOs closely monitored the progress and changes in the patient. In the beginning, SOs mentioned that they did not expect radical change. They looked for small progress and anticipated improvements in the patient’s ability to function. SOs stated that change in real-life situations was important to them. Small changes make a big difference. Having more interactions and seeing the patient become more independent are the first signs of progress.

“When I asked him what he wanted to eat, he answered. He didn’t do that before (ECT). Yesterday we looked at a television show and I commented on something. I was surprised because he reacted.”

“She put on her clothes last week. Didn’t wear her pyjamas all day.”

“When his friend visited, he (the patient) seemed to have a conversation with him. It was already a long time ago that that was possible.”

For SOs, changes in interactions, communication, and self-care were the first and most important indicators of the effect of ECT. They did not expect radical transformation but rather assessed progress by comparing small changes to the patient’s prior life, the life before the illness. Recovery, in their view, was marked by the patient “becoming his/her former self again”.

“When I arrived with my parents, mother (the patient) was cleaning the windows. Having a clean house and especially clean windows has always been very important to her. The disease made her indifferent to a clean house and seeing her cleaning the windows when I arrived, gave me the feeling that I had my mother back. It made me cry from relief, joy and tristesse for what she must have gone through (cries).”

SOs monitored effects and side effects almost constantly. As for side effects, they stated that they wanted to make sure ECT did not make the situation worse in the short term and that they also did not want to risk (brain) damage in the future. Memory loss is the side effect they monitored most closely. The side effect that was seen as most hindering in daily life was fatigue and the need for sleep after ECT.

“After ECT she sleeps for three days. She disappears in the bedroom. That is not good. It takes a week before she recovers a bit and then the next ECT is coming. This is no progress, on the contrary. If it stays like this, we will not go on with it (ECT).”

The effects and side effects are balanced. As long as the benefits outweigh the discomfort caused by the side effects, SOs perceived ECT as a valuable therapy. They actively encouraged and supported the patient in continuing ECT while also addressing practical and organizational challenges to ensure treatment adherence. The positive changes, consistently described as “the patient returning his/her old self again”, justify the significant efforts required to adjust daily life around ECT sessions.

As the patients showed improvement, SOs increasingly prioritized the patients’ own assessment and evaluation of the treatment. If patients were able to express discomfort and side effects of ECT, SOs followed and respected their judgment about whether to continue ECT. SOs refrained from pressuring them to proceed with ECT if they did not want to go on with it. Data indicate that SOs initially took on the responsibility of making decisions until the patients could express what they wanted. From that point on, SOs shifted from making decisions to advocating for the patients’ wishes. At that stage, they no longer assumed responsibility for the continuation or cessation of ECT but instead supported the patients in their own decisions.

“He says the ECT does something with him. He cannot say what exactly, but it does not feel good. He also complains of memory loss and that frightens him. He wants to stop ECT. Eventually it is he who must undergo the treatment every time. I can point out the positive effects, but I cannot decide what is bearable and good for him.”

### ECT as part of daily life

When confronted with maintenance ECT, SOs started integrating it into their daily routines. Recurrent ECT sessions require a feasible organization and practical arrangements that do not unnecessarily interfere with their core responsibilities and roles. SOs sought to establish a new routine and needed help from (mental) health professionals to adapt ECT into a life that could be considered “normal” again. Professionals who are willing to consider the needs of SOs in planning ECT, who facilitate the hospital stay during ECT, or who help find solutions for practical issues, such as transportation, timing of ECT, and food, are highly valued.

“As it is a long drive to bring my wife to the hospital for treatment, I cannot go to work the day of ECT. I have a busy job and can’t afford to stay home or work less. But if I can stay in a private hospital room where I can quietly work on my laptop while my wife gets ECT or recovers from it, I can do my job and stay with my wife at the same time. Some nurses really think of my needs and arrange a private room, others don’t and then it costs me a lot of energy to argue and find other solutions. I know my week will be a mess then.”

“ECT was always on a day that I had to work and I wasn’t allowed to change my work schedule. That was a real problem because in the long run I wouldn’t be able to keep my job. I also couldn’t find anyone else to accompany my son. When talking to the nurse about it, she arranged ECT-sessions on another day. That was such a relief and resolved a lot of my problems and worries for the future.”

#### The challenge of disclosure: overcoming stigma and rebuilding social connections

Beyond logistical and practical arrangements, integrating ECT into daily life also requires navigating social disclosure and talking about it with people in their environment. SOs reported avoiding discussions about ECT, even with close family members, both due to the patient’s condition and because of concerns about stigma. While they acknowledged that not talking about it could lead to social isolation, they felt reluctant to do so. Disclosure of ECT is perceived as a difficult but necessary hurdle to take in restoring social connections. When they did discuss ECT with family and friends, they frequently encountered misinformation and a lack of knowledge about ECT. As a result, SOs often felt the need to defend the decision to initiate ECT and reassure others that it is a legitimate treatment. They recognized the same fears and uncertainties in others they had experienced. It cost them energy to explain everything, but discussing their experiences fostered a renewed sense of connection and support in their social circles.

## Discussion

This study provides an in-depth exploration of the perspectives and experiences of SOs involved in ECT and highlights the importance of their involvement in ensuring successful treatment.

Much of the existing literature focuses on the concept of caregiver burden, recognizing that chronic mental health conditions place significant emotional, psychological, social, and financial strains on both patients and their informal caregivers (18, 19). While this study acknowledges the burden, it goes further by illustrating the specific challenges that SOs encounter in the context of ECT. Our findings align with prior qualitative research on the experiences of relatives of individuals with depression, particularly regarding emotional distress and interruptions to relationships (20). However, ECT introduces additional complexities as it is a particularly impactful treatment. Previous qualitative studies have reported similar distress and anguish because of the illness among families of ECT patients (21, 22). The notion of ECT as a “last resort” is mentioned along with patients expressing a sense of “blind trust” in the psychiatrist during the decision-making process toward ECT (21). However, our findings show that SOs actively engage in decision-making, seeking guidance, involvement, and reassurance from the psychiatrist—not just to make the decision but also to cope with its weight. Hope emerged as a central theme driving the decision to start and pursue ECT. While Sethi and Williams (22) described hope as a factor in families’ responses to ECT, they did not explicitly link it to the decision-making process. Our findings suggest that hope is crucial not only for initiating treatment but also for sustaining it. Previous studies on chronic illness have emphasized the role of hope in engaging with challenging treatments, such as cancer therapies (Snyder et al., 2002). In the context of ECT, SOs derive hope from the information provided by the psychiatrist, reinforcing the need for clinicians to communicate effectively and instill a realistic sense of optimism.

Hope can thus serve as both a therapeutic target and an integral part of informed consent, influencing adherence to therapy. Ensuring that SOs receive adequate, clear, and transparent information about ECT can strengthen their ability to balance risks and benefits, ultimately improving treatment adherence. Beyond the provision of information, clinicians may consider actively fostering hope and incorporating tailored interventions that support hope and optimism into the care of ECT patients and their SOs.

During the course of ECT, SOs closely monitor the real-life impact of ECT treatment. Unlike formalized assessments or rating scales that measure change over short periods of time, SOs evaluate progress in the broader context of the patient’s pre-illness functioning (23). This discrepancy between subjective and clinical assessments underscores the need for clinicians to recognize the limitations of rating scales and to incorporate not only the patient’s but also the SO’s perspectives in evaluating treatment outcomes.

A significant proportion of the sample was SOs of patients undergoing M-ECT. Existing literature underscores the essential role of M-ECT in sustaining remission and preventing relapse (11, 12). However, our study reveals that while SOs acknowledge the benefits of M-ECT, they also face logistical and emotional challenges in sustaining long-term adherence. Practical support from ECT clinics, such as accommodating schedules and providing adequate facilities, is crucial. These considerations, while seemingly peripheral, impact the overall experience and adherence of SOs to ECT.

Additionally, stigma remains a substantial burden for SOs; even within their own families, SOs report encountering skepticism and misinformation. This underscores the need for targeted informational programs by ECT clinics that extend beyond the patient–clinician relationship to SOs and their broader network. One potential intervention to mitigate stigma and strengthen social support is the organization of network-focused meetings, similar to those implemented in other medical contexts, such as adolescent and young adult cancer care (24). These meetings are organized for a patient and his/her SO. Meaningful people from their network are invited to the hospital to address concerns and receive validated information from healthcare professionals, ultimately fostering a supportive environment for both patients and their SOs.

### Limitations

This study has some limitations. It was monocentric, conducted in one supra-regional hospital. Although the hospital treats patients from different regions, psychiatrists, and other hospitals, and although the themes are not explicitly or exclusively linked to the hospital or psychiatrists’ practice, there may be bias or lack of diversity. This possibly reduced the transferability of the findings. To capture diverse perspectives, we included SOs of patients who had just started ECT along with those receiving M-ECT. However, since all interviews were conducted post-ECT, there is a potential for recall bias, and the timing of the interviews may have influenced the responses. Interviews conducted shortly after the acute phase may have reflected more distress, while later interviews may have been shaped by perceived improvement. While these factors introduce variability, the consistency of themes across participants suggests that the risk of systematic bias was limited. There were no SOs of patients who dropped out, refused, or did not start ECT. This could emphasize the more positive perceptions and themes. A more heterogeneous selection of SOs, e.g., from patients who dropped out or did not start ECT, would be desirable to gain broader insight. Additionally, there was only one participant under the age of 56. Young SOs were underrepresented in this study, raising the question of whether the process differs for younger individuals. Additionally, the sample primarily consisted of SOs of patients with affective disorders and no other psychiatric disorders, such as schizophrenia. As this is a qualitative and explorative study, it is not possible to compare experiences between groups or analyze covariates such as phase of ECT, illness severity, duration of illness, or relation to the patient.

### Implications for practice

The findings of this study highlight five important key areas where clinical practice can be improved to better support SOs throughout the ECT treatment.

#### Encouraging a nuanced approach to shared decision-making

SOs often experience a heavy burden and responsibility in making treatment decisions. This study highlights the importance of a balanced and supportive approach in which clinicians actively assist and guide SOs, ensuring that they do not feel solely responsible. At the same time, SOs play a critical role in evaluating treatment outcomes. Their perspective and real-world observations provide valuable insights that extend beyond standardized assessment tools.

#### Providing tailored and experience-based information to foster hope and perspective

SOs rely on psychiatrists not only for medical facts but also for personalized, experience-driven guidance that addresses their specific concerns. While supplementary materials such as brochures, videos, and testimonials can help, they cannot replace a psychiatrist’s tailored explanation that fosters trust, reduces uncertainty, and supports informed decision-making. Beyond factual knowledge, the way psychiatrists communicate information—highlighting realistic hope and treatment potential—plays a crucial role in how SOs perceive and engage with ECT. Ensuring that SOs receive clear, transparent, and empathetic communication can strengthen their confidence in treatment decisions, ultimately improving adherence and emotional resilience.

#### Facilitating practical solutions

Logistical challenges, such as scheduling and transportation, can affect the continuation of maintenance ECT. Instead of one-size-fits-all solutions, providing tailored practical support that meets the specific needs of SOs can make a meaningful difference in ensuring treatment adherence.

#### Developing strategies to destigmatize ECT and psychiatric illnesses

To combat the stigma surrounding ECT and psychiatric illnesses, targeted initiatives could be implemented. These may include organizing network meetings and multi-family meetings and increasing public engagement through media and community outreach efforts.

### Recommendations for future research

Future research could focus on exploring the perceptions of young SOs or SOs of patients who did not start or dropped out of ECT. Their perceptions and the dynamics between them and the patient could give valuable insights. The study highlighted specific perspectives on the role of the psychiatrist. Further research can elaborate on the key components of this role. Studying the function of hope before and throughout the process of ECT, mental illness, and recovery can help to understand and guide SOs and patients. Research on the role of SOs in assessing the effects and side effects of ECT and in defining (intermediate) goals and outcomes could be valuable to complement clinical evaluation (instruments). In this study, the focus was on the perspectives of SOs. It may also be relevant to explore how patients, psychiatrists, and other mental healthcare professionals perceive and experience ECT and how interpersonal dynamics and boundaries influence the decisions course of the treatment.

## Conclusion

This study gives additional insight into the perspectives of SOs of patients before, during, and after ECT. Despite experiencing strain on multiple levels, SOs seek information, understanding, and hope before the initiation of ECT. They express a desire to be involved in evaluating the treatment and encounter various practical challenges during maintenance therapy. Our findings highlight the necessity of an integrated and holistic approach to ECT care, one that actively includes SOs as key stakeholders in the treatment process.

## Data Availability

The datasets presented in this article are not readily available because the Dataset was anonymised qualitative data from interviews. Requests to access the datasets should be directed to pieter-jan.geerts@azgroeninge.be.
